# NET degradation attenuates ricin-induced acute lung injury and protects mice from ARDS

**DOI:** 10.1186/s10020-025-01370-8

**Published:** 2025-09-29

**Authors:** Anita Sapoznikov, Yentl Evgy, Liat Fux, Ilya Ruderfer, Yakir Nataf, Yael Hayon, Shay Zamin, Roey Mizrachi, Rachel Pessah, Yoav Gal, Alon Ben-David, Noam Erez, Reut Falach

**Affiliations:** 1 Department of Biochemistry and Molecular Genetics, Institute for Biological Research, Ness Ziona, Israel; 2 Protalix Biotherapeutics, Carmiel, Israel; 3 Department of Biotechnology, Institute for Biological Research, Ness- Ziona, Israel; 4 Department of Infectious Diseases, Institute for Biological Research, Ness-Ziona, Israel

**Keywords:** Ricin, NETosis, NETs, Lung, PAD4, CitH3, DNase I, ARDS

## Abstract

**Background:**

Neutrophils are critical first responders of the innate immune system, rapidly recruited to sites of infection or sterile injury. Upon activation by pathogen- or damage-associated molecular patterns, neutrophils initiate antimicrobial responses, including cytokine release, phagocytosis, and the formation of neutrophil extracellular traps (NETs). While NETosis plays a protective role, excessive NET formation can exacerbate inflammation and tissue damage. Pulmonary exposure to ricin, a potent toxin derived from *Ricinus communis*, results in acute lung injury characterized by neutrophil infiltration, cytokine production, vascular leakage, and pulmonary edema. This study investigated the contribution of NETosis to ricin-induced lung pathology and explored the therapeutic potential of targeting NETosis with a long acting recombinant DNase I (PRX-119) to attenuate lung injury.

**Methods:**

CD1 outbreed mice were pulmonary exposed to ricin, and bronchoalveolar lavage fluid (BALF) and lung tissues were collected at various time points post-exposure. NETosis was assessed by immunofluorescence and Western blot analysis of markers, including peptidyl arginine deiminase 4 (PAD4), citrullinated histone H3 (citH3) and myeloperoxidase (MPO). Therapeutic intervention included administration of a NET-degrading DNase agent in combination with an anti-ricin antibody. Cell-free DNA levels, NETosis markers, neutrophil infiltration, lung histopathology, vascular permeability and the expression of pro- and anti-inflammatory mediators were evaluated. Weight loss and survival were also monitored and compared between anti-ricin monotherapy and combined anti-ricin and plant-produced human recombinant long acting (LA) DNase I (PRX-119), a novel NET degradation therapy.

**Results:**

Ricin exposure led to elevated pulmonary levels of PAD4, citH3 and MPO, accompanied by extensive NET formation in both BALF and lung tissue. Mice receiving combined therapy with a newly developed DNase I - based agent (PRX-119) and an anti-ricin antibody treatment exhibited significantly improved survival and reduced weight loss compared to antibody monotherapy. The combined treatment not only significantly reduced NETosis markers, but also improved lung histopathology, reduced vascular leakage and pulmonary edema and altered the levels of proteins involved with pro- or anti-inflammatory effects, Dkk-1, CD93 and Periostin.

**Conclusions:**

These findings demonstrate for the first time that NETosis plays a significant pathological role in ricin-induced lung injury. Moreover, they underscore the therapeutic potential of combining advanced NET-degrading agents, specifically PRX-119, an advanced DNAse I under development, with toxin-neutralizing antibodies as a promising strategy to reduce acute lung damage and enhance clinical outcomes.

**Supplementary Information:**

The online version contains supplementary material available at 10.1186/s10020-025-01370-8.

## Introduction

Neutrophils are essential effector cells of innate immunity and key regulators of both innate and adaptive immune responses. They are produced in the bone marrow and represent the most abundant immune cell in the human blood. Neutrophils patrol blood vessels and in case of infection or sterile injury, are among the first immune cells recruited to the inflamed tissue (Stark et al. 2013). Neutrophils are activated through receptors that recognize proinflammatory signals such as pathogen-associated molecular patterns (PAMPs), which originate from an invading pathogen, or damaged-associated molecular patterns (DAMPs) that derive from the host (Pittman and Kubes 2013). Activation of neutrophils triggers a signaling cascade that induces a variety of effector functions. Besides releasing cytokines and chemokines to maximize the host’s immune response, neutrophils also exert three major antimicrobial effector functions: release of granules that are packed with antimicrobial peptides, internalization and neutralization of pathogens by phagocytosis, and release of neutrophil extracellular traps (NETs) (Poli and Zanoni 2023). To maintain tissue homeostasis, neutrophil responses must be commensurate to the threat, in order to effectively eliminate an invading pathogen, without damaging the host (Brinkmann et al. 2004). The production and release of NETs, known as NETosis, was first described as a tool used by neutrophils to kill bacteria extracellularly that may or may not require neutrophil death (Yipp and Kubes 2013). NETs are long filamentous extracellular web-like structures of decondensed DNA, which are mainly released from the nucleus of cells that produce them. They are characterized by the presence of citrullinated histone H3 and proteins that are usually stored in azurophilic granules, such as neutrophil elastase (NE), cathepsin G and myeloperoxidase (MPO) (Poli and Zanoni 2023). One of the best-described pathways that lead to NETosis involves production of reactive oxygen species (ROS) via NADPH oxidase. ROS stimulate MPO, enable translocation of the NE from azurophilic granules into the cytosol, and then into the nucleus, where it starts to degrade histones, unpacks the chromatin, and releases DNA. ROS also activate peptidyl -arginine deiminase 4 (PAD4), a key enzyme for the induction of NETs (Wang et al. 2009; Li et al. 2010; Lewis et al. 2015), which converts the arginine on histones to citrulline. This results in the loss of a positive charge and facilitates chromatin decondensation. PAD4 also achieves DNA decondensation by decreasing the affinity between DNA and histones (Rohrbach et al. 2012). Decondensation of DNA and its expansion then leads to nuclear membrane disruption. Chromatin is released into the cytosol, where it is decorated with a variety of granular and cytosolic proteins (Papayannopoulos 2018). The release of nuclear DNA follows the rupture of the plasma membranes and results in death of the neutrophil (Pilsczek et al. 2010). NETosis was first described as an antimicrobial mechanism for entrapping and killing bacteria, but NETs are also induced by a large variety of stimuli (Branzk and Papayannopoulos 2013) that include fungi (Smolarz et al. 2021) and viruses (Saitoh et al. 2012; Veras et al. 2020; Koupenova et al. 2019), as well as parasites (Abi Abdallah et al. 2012). In addition, NETosis can occur during noninfectious sterile inflammation (Huang et al. 2015; Jorch and Kubes 2017), and are also involved in the development of immune-mediated disorders (Caudrillier et al. 2012; Liu et al. 2018; Khandpur et al. 2013; Domizio and Gilliet 2019; Chatfield et al. 2018) and genetic disorder, such as Cystic Fibrosis (Manzenreiter et al. 2012). Interestingly, lungs have a high availability of oxygen that can be transformed into ROS, making this tissue particularly susceptible to the detrimental effects of uncontrolled NETosis. This is a factor that contributes to the pathogenesis of numerous chronic inflammatory lung diseases, such as cystic fibrosis, chronic obstructive pulmonary disease (COPD) and allergic asthma (Liu et al. 2016). Lastly, it has been demonstrated that during lung infection with SARS-CoV-2, induction of NETosis is a key feature of severe COVID-19 (Veras et al. 2020).

The mechanisms responsible for clearing NETs remain poorly defined. During infection, NETs can persist for several days and are believed to be degraded primarily by the plasma nuclease DNase I (Papayannopoulos 2018). Evidence indicates that NETs can inflict tissue damage during both infectious and sterile inflammatory conditions by directly killing epithelial and endothelial cells (Wilhelmsen and Pitt 1996; Pincus et al. 2015). For instance, in fungal pulmonary infections, excessive NETosis has been shown to injure the lung epithelium (Gal et al. 2017) and in transfusion-related acute lung injury (TRALI), NETs have been implicated in endothelial damage (Katalan et al. 2017). NET-derived histones are cytotoxic due to their ability to disrupt cell membrane integrity (Sapoznikov et al. 2019; Zemans and Matthay 2017). Additionally, other proteins associated with NETs, such as defensins, can permeabilize eukaryotic cell membranes (Gal et al. 2014), while NE degrades extracellular matrix proteins, disrupting cell–cell junctions (Falach et al. 2018). Pulmonary exposure to the castor oil plant-derived toxin ricin induces damage that is primarily localized to the lungs and is characterized by prominent interstitial pneumonia, accompanied by the release of pro-inflammatory cytokines, massive neutrophil infiltration, increased vascular permeability, edema, and hemorrhages. Ultimately, the extensive pneumonia, which manifests as severe cellular infiltration and excessive accumulation of pleural fluids, leads to respiratory failure and death. This pulmonary damage is classified as acute lung injury (ALI), which progresses to acute respiratory distress syndrome (ARDS) (Wilhelmsen and Pitt 1996; Pincus et al. 2015; Gal et al. 2017; Katalan et al. 2017; Sapoznikov et al. 2019). Neutrophil infiltration into the inflamed lung is a hallmark of various ARDS-related conditions, and the number of these cells directly correlates with disease severity (Zemans and Matthay 2017). We have previously demonstrated that a vast number of neutrophils are recruited to the lungs following ricin exposure and secrete matrix metalloproteinases (MMPs), which degrade different types of junctional proteins, including adherens-, tight-, and gap- junctions. These proteins are critical for maintaining the structural integrity of tissue by anchoring adjacent cells one to another. The breakdown of endothelial and epithelial junctional proteins leads to increased alveolar-capillary permeability, resulting in severe pulmonary edema and fatal respiratory failure due to fluid accumulation in the lungs. Specific depletion of neutrophils or inhibition of MMP activity in ricin-intoxicated mice reduced junctional protein damage, alleviated pulmonary edema, and significantly prolonged the mean time to death (Sapoznikov et al. 2019).

In the current study we investigated whether the extensive neutrophil recruitment to lung tissue in response to ricin exposure leads to the formation of NETs, and whether this process contributes to disease pathology. Furthermore, we examined the pathological consequences of NETosis by selectively inhibiting it, with the goal of determining whether targeted degradation of NETosis can alleviate disease symptoms, reduce lung tissue damage, and improve prognosis. Additionally, we evaluated whether combining NET-degrading therapy with a specific anti-ricin treatment could improve survival in exposed mice.

## Materials and methods

### Animals

CD1 outbred mice (females weighing 27–32 g) were purchased from Charles River Laboratories (England) and were kept in an animal husbandry facility for 4–8 days prior to commencement of the experiments. The animals were kept under a 12-hour light regime and had free access to food and water. The experimental protocols were approved by the Institutional Animal Care and Use Committee (IACUC) of the Israel Institute for Biological Research (protocols M-16-2023, M-12-24).

### Preparation of ricin toxin

The use of ricin in the current study and purification of ricin from castor beans were conducted under the safety and environmental regulations of the Israel Institute for Biological Research, in compliance with the Israeli law.

Crude ricin was prepared from seeds of endemic *Ricinus communis* as previously described (Gal et al. 2014). Shortly, seeds were homogenized in a blender (Waring, Torrington, CT) in 5% acetic acid (Merck, Darmastadt, Germany)/PBS (Biological Industries, Beth-Haemek, Israel). The homogenate was centrifuged, and the clarified supernatant containing the toxin was subjected to ammonium sulfate (Merck, Darmastadt, Germany) precipitation (60% saturation). The precipitate was dissolved in PBS and dialyzed extensively against the same buffer. The toxin preparation appeared on a Coomassie blue (Bio-Rad, Rishon Le Zion, Israel)-stained nonreducing 10%polyacrylamide gel (ThermoFisher Scientific, Carlsbad, CA) as two major bands of molecular mass of ~ 65 kDa (ricin toxin, ~ 80%) and 120 kDa (Ricinus communis agglutinin, ~ 20%). Protein concentration was determined as 2.86 mg/ml by 280 nm absorption (Nanodrop 2000; ThermoFisher Scientific).

### Production of PRX-119

PRX-119 is a PEGylated plant-produced long-acting recombinant human DNase I. PRX-119 is produced in two steps, the non-PEGylated DNase I is produced using the Protalix Biotherapeutics plant cell-based expression system ProCellEx^®^ followed by PEGylation with 5 kDa PEG-aldehyde. This technology is covered under patents WO 2013/114,374 and WO 2022/074656.

### Mice intoxication and treatment

Mice were anesthetized by intraperitoneal injection of 0.2 ml of ketamine (1.9 mg/mouse, Vetoquinol, Lure, France) and xylazine (0.19 mg/mouse, Eurovet Animal Health, AD Bladel, The Netherlands). Crude ricin was administered intranasally at a dose of 9.6 µg/kg body weight (2LD_50_), at a volume of 25 µl per nostril.

Anti-ricin antibody treatment was administered by single intravenous administration (100 µl), 24 h after exposure to ricin. The antibody was purified from hyperimmune plasma of horses vaccinated with ricin (RR003, a purified F(ab)_2_ antibody fragment, with a titer of 1716 Neutralizing Israeli Units) (Falach et al. 2018).

PRX-119 was administered intraperitoneally 5 mg/kg at a volume of 100 µl. Treatment commenced 24 h after exposure to ricin and was administered once a day for 14 days.

### Quantification of cytokines and lung damage mediators in BALF

BALF was collected by instillation of 1 ml PBS at room temperature via a tracheal cannula, and centrifuged at 240 g at 4 °C for 5 min. Supernatants were collected and stored at − 20 °C until further use. Interleukin-6 (IL-6), granulocyte colony-stimulating factor (G-CSF), keratinocyte chemoattractant (KC), macrophage inflammatory protein-2 (MIP-2), Interleukin-1β (IL-1β), monocyte chemoattractant protein (MCP-1), vascular endothelial growth factor (VEGF) and tumor necrosis factor-α (TNF-α) were quantified using ELISA kits (R&D Systems, USA), following the manufacturer’s instructions. Tailor-made Luminex mouse discovery assay was performed for measurement of the following mediators: C1qR1/CD93, CCL11/Eotaxin, CCL22/MDC, Chitinase 3-like 1/YKL-40, CXCL16, HGF, IL-1α/IL-1F1, IL-10, IL-13, MMP-9, Osteopontin, Periostin/OSF-2, Resistin, S100A9, Serpin E1/PAI-1, Thrombospondin-4, TIMP-1, Dkk-1, EMMPRIN/CD147 and LIX (R&D Systems, Biotest, USA).

### Protein quantitation

Protein concentration in BALF was determined using Bradford reagent. Volume of 20 µl of BALF from each sample were loaded and separated on NuPage 4–12% Bis-Tris SDS-PAGE Gel (Invitrogen, NP0335BOX) and transferred to an iBlot Transfer Stack Nitrocellulose PVDF membrane (Invitrogen, IB01001). The membranes were blocked for one hour in room temperature in TBST solution (0.15 M NaCl, 0.05% Tween-20, 0.01 M Tris-HCl pH 8) containing 5% Skim Milk powder (Difco Skim Milk, BD 232100). The incubation with rabbit primary antibody against each of the tested proteins (anti-citH3, ab5103; anti-PAD4, ab214810, anti-MPO, ab208670, Abcam) was performed for 16 h at 4 °C with gentle agitation. After washing with TBST, the membranes were incubated for one hour at room temperature with goat anti-rabbit IRDye 680RD secondary antibody (Li-COR, 926−68071) diluted 1:10,000 in TBST solution with 5% skim milk powder. After washing, the bands were visualized using ODYSSEY CLx (LI-COR) imager. The intensity of each band was quantified using ImageJ software. Quantitation of citH3 in BALF was also performed using ELISA kit for citrullinated histone H3 (Cayman Chemicals, 501620, clone 11D3).

### Quantification of cell-free DNA in BALF

Cell-free DNA quantification was performed using Quant-iT™ PicoGreen™ dsDNA (Invitrogen) kit in accordance with manufacturer’s instructions. Briefly, a calibration curve was prepared using quantities from 0 to 1000 ng/ml DNA in x10 intervals. Then, 100 µl volumes of BALF samples were transferred into black 96 well plates (Greiner) and an equal volume of PicoGreen dsDNA reagent was added to each well. Fluorescent reads of the DNA were measured in a Microplate reader (Gen 5 3.04), excitation and emission at 485 nm and 538 nm, respectively.

### Visualization of NETs in neutrophils isolated from BALF

One day before staining, round glass coverslips were inserted into 24 well plates and coated with 400 µl poly-L-lysine (ScienCell #0413) at a concentration of 10 µg/ml for one hour in at 37 °C. Subsequently, the wells were washed with double distilled water (DDW) and left to dry in air. BALF obtained from naïve or intoxicated animals was centrifuged and the cell pellet was separated and resuspended in an Opti-MEM medium containing 2% heat-inactivated Fetal Bovine Serum (FBS, Sigma-Aldrich). The resuspended cells were loaded on the coverslips for one hour at 37 °C to allow them to adhere. The cells were fixed with 4% paraformaldehyde for 15 min at room temperature and then dried. Next, the cells were permeabilized with 0.5% Triton X-100 in PBS for 10 min at room temperature and blocked with 5% BSA in PBS for 40 min at room temperature. Cells were stained with primary antibody (anti-citH3, ab5103; or anti-MPO, ab208670, Abcam) resuspended in TBS containing 5% BSA and incubated over night at 4 °C. The next day, the cells were washed twice with TBS, incubated with anti-rabbit Alexa Fluor 594 secondary antibody (Invitrogen) for one hour at room temperature, and washed twice with TBS. For staining of extracellular cell free DNA, the cells were incubated with Sytox Green (Invitrogen) diluted 1:25,000 in PBS for 10 min at room temperature. The dye was then washed using TBS and nuclear staining was performed using Prolong^®^ Gold antifade reagent containing DAPI (Molecular probes).

### Visualization of NETs in neutrophils isolated from lungs

Lungs were harvested, cut into small pieces and digested by incubation with 4 mg/ml Collagenase D (Roche, Mannheim, Germany) in PBS Ca^+2^ Mg^+2^ (Biological Industries, Beit Haemek, Israel) for 2 h at 37 °C. The tissue was then meshed through a 70 μm cell strainer. Neutrophils were isolated using a Histopaque gradient. Briefly, 3 ml Histopaque-1119 (Sigma) was inserted into 15 ml tubes and on top of it, 3 ml Histopaque-1077 (Sigma) was carefully layered. Above the gradient, 6 ml of lung cells suspended in PBS containing 5% BSA were cautiously added. The tubes were centrifuged at 700 g for 30 min at room temperature without brake and the neutrophils which resided in the interface between the gradient layers, were carefully collected. Neutrophils were isolated by negative selection kit (Miltenyi Biotec, 130-097-658). The isolated cells were counted and resuspended in Opti-MEM medium containing 2% heat-inactivated FBS. Then, 1–2 × 10^6^ cells were loaded on poly-L-Lysine coated glass coverslips in a 24 well plate. Subsequent preparations and NETs staining were performed as described above with respect to staining BALF neutrophils.

### Histology and immunofluorescence

To visualize NETosis in lung tissue, lungs were collected and fixed in 4% buffered formaldehyde in PBS pH 7.2–7.4 (Bio Lab, Israel) for 2 weeks. Sections of 5 μm were prepared after paraffin embedding and using a microtome (RM 2255, Leica, Germany). The sections were deparaffinized and antigens were exposed by incubation with Target Retrieval Solution (DAKO) at 95 °C for 30 min. After blocking with 5% BSA in PBS, the slides were incubated with the primary antibodies rabbit anti-citH3 (ab5103, 1:200), and rat anti-Ly6B (ab53457, 1:200) (Abcam) over night at 4 °C. Subsequently, the slides were incubated for 1 h at room temperature with fluorescent secondary antibodies, Alexa Fluor 594-coupled goat anti-rat IgG (Abcam, ab150160, 1:100) and Atto550 goat anti-rabbit IgG (Rockland, 611-154-122s, 1:200). Nuclear staining was performed with Prolong^®^ Gold antifade reagent containing DAPI (Thermo Fisher Scientific). Image acquisition was performed using LSM 710 confocal microscope (Zeiss, Germany) with the following lasers argon multiline (458/488/514 nm), diode 405 nm, DPSS 561 nm and helium-neon 633 nm (objective lens LD C-Apochromat 40×/1.1 W Korr M27, image depth 8-bit, zoom 0.6, filters 410–521, 502–629, 570–609, 604–733 nm for DAPI, Sytox Green, citH3 and Ly6B, respectively). Analysis of the mean fluorescence intensity (MFI) of the staining was acquired using Zen 2008 (Zeiss, Germany).

To examine the lung morphology, tissue sections were stained with Hematoxylin and Eosin (H&E). The stained sections were photographed using 3D HISTECH Panoramic Midi II slide viewer (Budapest, Hungary), and analysis was performed using Case Viewer 2.4.0 software (Budapest, Hungary). Semi-quantitative assessment of the histological injury score in the lungs of mice was performed according to established published criteria: inflammatory cells, hyaline membrane, thickening of alveolar wall, enhanced injury, vessel congestion, and atelectasis (Silva et al. 2022). These parameters provided a score of 0 to 8, where 0 means “no phenomenon” and 8 means a severe phenomenon. Lungs were obtained from 5 mice in each treatment group. The score for each parameter was calculated as the average of five different fields. The percentage of the histological injury score for each animal was determined using the following equation: (sum of scores for the six evaluated parameters/48) × 100, where 48 is the maximum possible score.

### Lung permeability analysis

Lung permeability was determined by the Evans Blue dye (EBD) extravasation method as follows: 7.5 mg/ml EBD (Sigma-Aldrich) was injected intravenously at a dose of 50 mg/kg, and allowed to circulate for 1 h. Mice were then anesthetized, and the lungs were perfused by cutting the left atrium and flushing with 5 ml PBS through the right ventricle. The lungs were removed and EBD was extracted by incubation of the tissues in 0.5 ml of formamide (Sigma-Aldrich) at 60 °C for 24 h. EBD optical density in the supernatant was measured at 620 nm in a Spectramax ABS Plus (Molecular Devices) spectrophotometer.

### Statistical analysis

All statistical analyses were conducted with GraphPad Prism software (version 5.01, GraphPad Software Inc., La Jolla, CA, USA, 2007). Data were first tested for normality using the Shapiro–Wilk test. Simple comparisons were performed using the unpaired two-tailed Student’s t-test. For multiple comparisons, one-way or two-way analysis of variance (ANOVA) tests followed by Tukey’s or Bonferroni multiple comparisons test were applied, respectively. Survival data were analyzed using Kaplan–Meier survival curves, and differences between groups were assessed with the Log-rank (Mantel–Cox) test. Data are presented as means ± SEM. Differences were considered significant at *p* < 0.05.

## Results

### Assessment of NETosis in the lungs of mice following pulmonary exposure to ricin

**Fig. 1:**
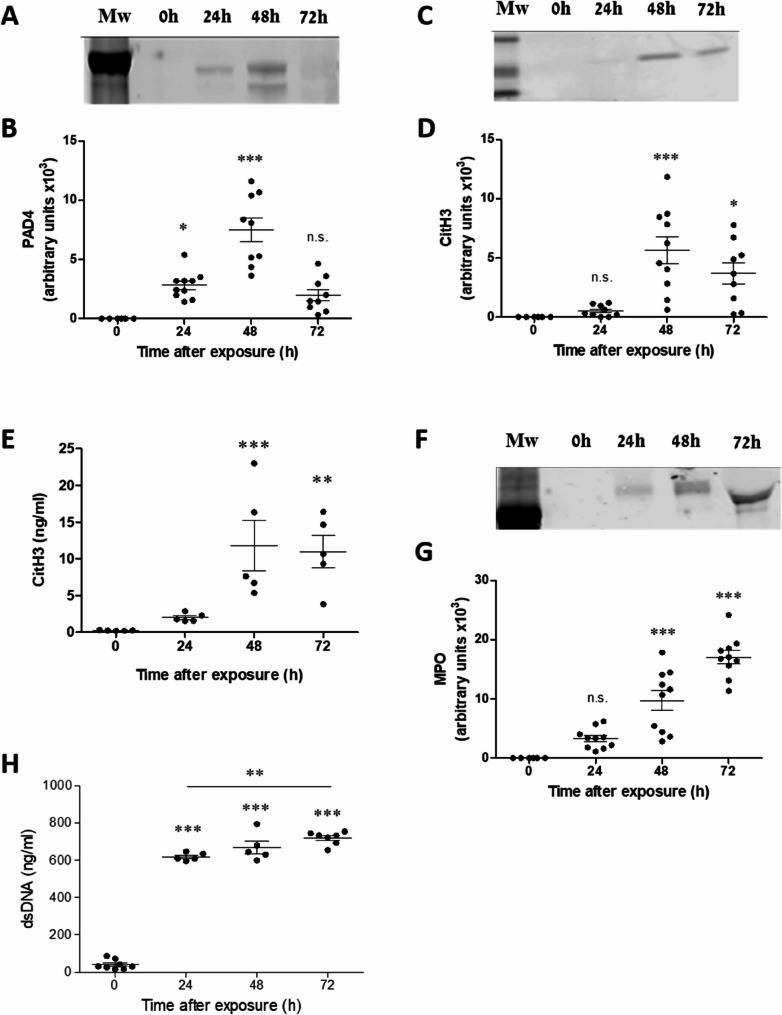
Expression levels of PAD4, citH3, and MPO in BALF of mice following ricin exposure. Western blot analysis of PAD4 (**A**), citH3 (**C**), and MPO (**F**) in BALF collected at different time points from control mice and mice intranasally exposed to 9.6 µg/kg (2LD_50_) ricin. Densitometric quantification of PAD4 (**B**), citH3 (**D**), and MPO (**G**) was performed using ImageJ software. **E** citH3 levels in BALF were further quantified using a commercial ELISA assay. **H** Quantification of extracellular DNA levels in BALF of ricin-exposed mice at different time points following ricin exposure. Data are presented as mean ± SEM (*n* = 5–10 per group, each point on the graph represents an individual mouse). Statistical significance: *p < 0.05, **p < 0.01, ***p < 0.001 compared to non-exposed control mice. (n.s., not significant)

While NETosis originally evolved to eliminate pathogens, it also occurs during sterile inflammation, where excessive neutrophil activation can cause tissue damage (Kovtun et al. 2018). In the lungs, NETs compromise the alveolar-capillary barrier, increasing permeability and leading to hemorrhages (Narasaraju et al. 2011). Following pulmonary exposure to ricin, extensive neutrophil infiltration has been observed constituting nearly half of all lung cells at certain time points post-exposure (Sapoznikov et al. 2015, 2019). To determine whether these pulmonary neutrophils undergo NETosis, we intranasally exposed mice to ricin and collected BALF at 24, 48, and 72 h post‑exposure. The presence and levels of PAD4, citH3, and MPO which are the hallmarks of NETosis, were assessed in BALF by Western blot analysis. Results revealed a significant increase in PAD4 levels in BALF at 24 h post-exposure, reaching peak levels at 48 h, followed by a decline at 72 h (Fig. 1A, B). CitH3 levels peaked at 48 h and remained high at 72 h (Fig. 1C, D), consistent with ELISA measurements of citH3 (Fig. 1E). Similar kinetics were observed for MPO (Fig. 1F, G). The presence of PAD4, citrullinated histones and extracellular MPO in BALF indicates that NETosis occurred in the lungs following exposure to ricin.

**Fig. 2:**
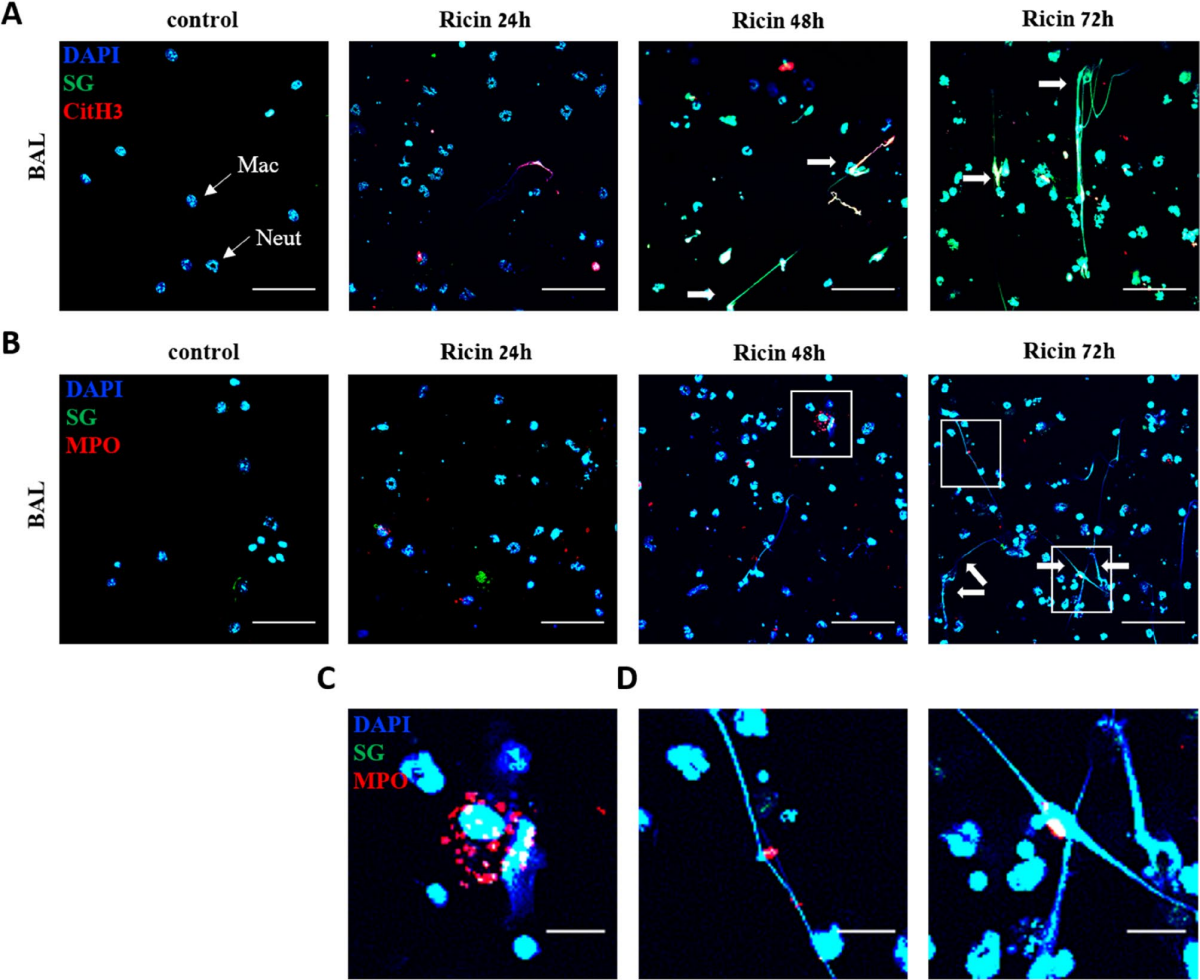
In situ NET formation by neutrophils from BALF of mice following ricin exposure. **A** Representative images of neutrophils from BALF undergoing NETosis. Staining for citH3 (red), extracellular DNA (SG, green), and nuclear staining with DAPI (blue) was shown. Samples were collected at different time points from control mice and mice intranasally exposed to 9.6 µg/kg (2LD_50_) ricin. Scale bar: 50 μm. In control BALF samples, both alveolar macrophages (MAC) and neutrophils (Neut) were observed (thin arrows). Following ricin exposure, alveolar macrophages were depleted, leaving neutrophils as the predominant cell type. NET structures, extended chromatin fibers associated with neutrophils stained in red, green, and blue, were most prominent at 48- and 72-hours post-exposure (bold arrows). **B** Representative images of MPO (red) staining in BALF neutrophils from control and ricin-exposed mice at different time points. Extracellular DNA was stained in green, and nuclei were stained with DAPI (blue). Scale bar: 50 μm. Enlarged views of the regions outlined by rectangles in the BALF at 48 h (**C**) and 72 h (**D**) post-ricin exposure show MPO staining (red dots) either not associated with NET structures (green and blue) (**C**) or decorating NETs (**D**), respectively. Scale bar: 10 μm

In addition, the level of cell-free DNA was quantified at different time-points following ricin exposure using Sytox Green dye. This dye intercalates into DNA that is accessible outside of intact cells, such as that released during NETosis or from dead/dying cells. The analysis showed that of cell-free DNA levels were already elevated 24 h post-ricin exposure and continued to raise at 48 and 72 h (Fig. 1H). Importantly, Sytox Green does not distinguish the source of extracellular DNA, which may originate from NETs, as well as from apoptotic or necrotic cells. The latter are abundant in the lung following ricin intoxication, owing to direct ribosome inactivation and inhibition of protein synthesis that ultimately leads to cell death (Wilhelmsen and Pitt 1996; Sapoznikov et al. 2015). Therefore, we combined Sytox Green (SG) staining with NET-specific markers to more precisely determine the contribution of NETs to extracellular DNA. To directly visualize NET formation in situ, BALF samples were analyzed for extracellular DNA and citH3 staining. Control samples displayed intact alveolar macrophages and neutrophils, whereas in BALF samples from exposed mice, only neutrophils were observed (Fig. 2A) due to rapid alveolar macrophage depletion following intoxication (Sapoznikov et al. 2015). At 24 h post-exposure, citH3-positive staining was detected, but characteristic NET structures of extracellular DNA were not yet visible. By 48 and 72 h, thread-like extracellular structures positive for citH3, extracellular DNA staining and extending from neutrophils were observed, indicating widespread NET formation (Fig. 2A). MPO staining revealed that the enzyme was released from neutrophils at 24–72 h post-exposure, but its distribution was not restricted to NETs, sometimes appearing as dispersed punctate staining near neutrophils (Fig. 2C, Ricin 48 h) rather than being associated with chromatin networks (Fig. 2D, Ricin 72 h). Therefore, we selected a combination of citH3 and extracellular DNA staining as markers of NETs throughout this study.

**Fig. 3:**
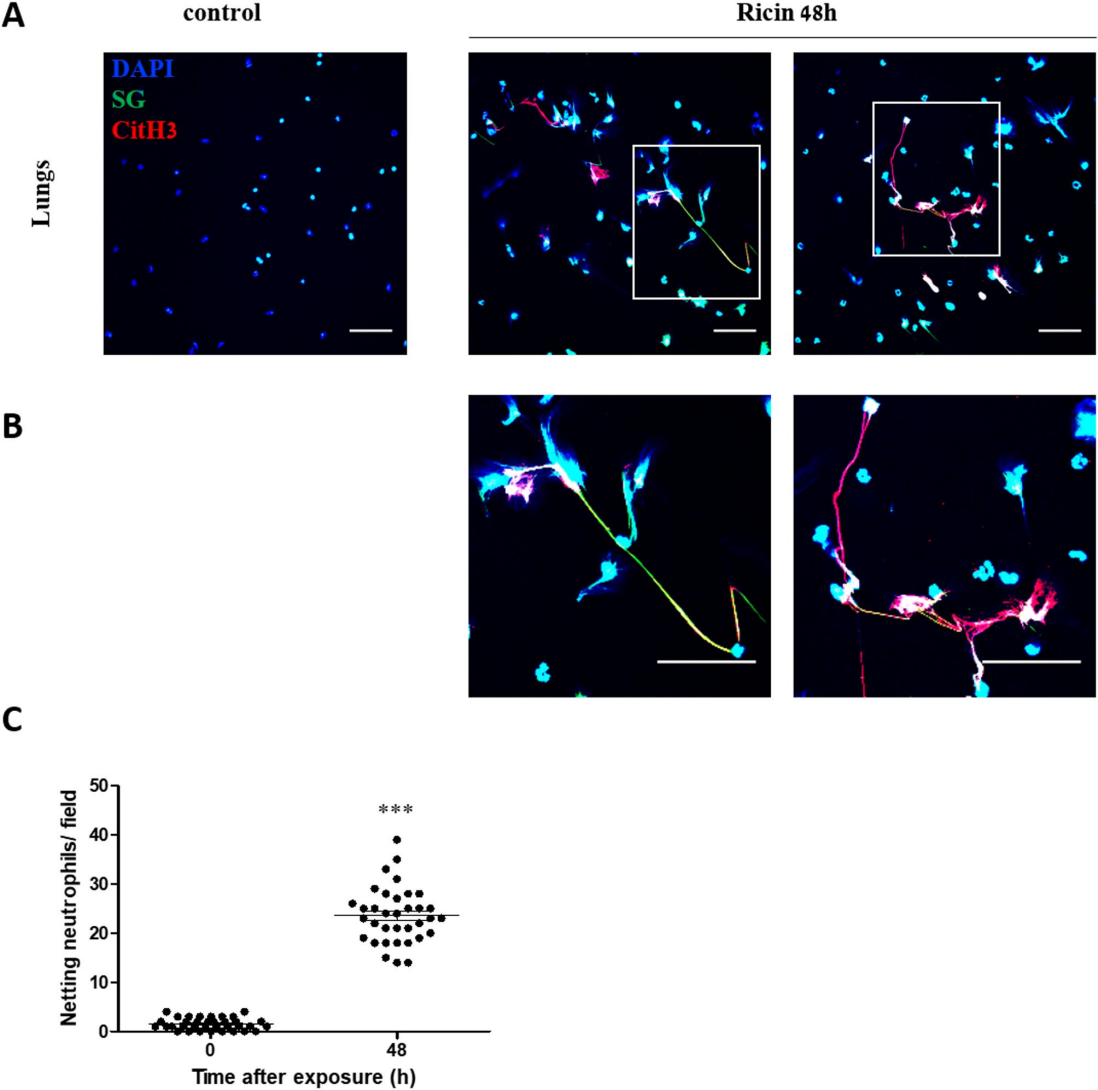
In situ NET formation by neutrophils isolated from the lungs of mice following ricin exposure. **A** Representative images of neutrophils undergoing NETosis, isolated and purified from the lungs of control mice or mice intranasally exposed to 9.6 µg/kg (2LD_50_) ricin at 48 h post-exposure. Staining for citH3 (red), extracellular DNA (green), and nuclear staining with DAPI (blue) was shown. Scale bar: 50 μm. **B** Enlarged views of the regions outlined by rectangles in **A** show NET structures appeared as long chromatin threads extending from neutrophils and decorated with citH3. Scale bar: 50 μm. **C** Quantification of NETosis by counting the number of neutrophils undergoing NET release per microscopic field. Five randomly selected fields were imaged per sample (*n* = 5) and the number of NET-releasing neutrophils was counted in each field. Each data point represents the number of neutrophils undergoing NETosis within a given field. ***p < 0.001 compared to non-exposed control mice

Since BALF neutrophils represent only a subgroup of total lung neutrophils, we next examined NETosis in neutrophils isolated directly from lung tissue. Neutrophils were enriched using density gradient centrifugation and isolated by magnetic sorting at 48 h post-exposure, the timepoint of peak NETosis, as was shown in Fig. 1. Isolated neutrophils were stained for extracellular DNA and citH3, revealing that lung-resident neutrophils underwent NETosis similar to BALF neutrophils, with characteristic extracellular chromatin networks (Fig. 3A, B). To ensure that the observed NETosis was not an artifact resulting from neutrophil activation during the isolation process, we quantified NETosis (extracellular DNA filaments positive for citH3) and compared the number of NET-forming neutrophils in both control- and ricin-intoxicated mice 48 h post-exposure. Quantification of NETosis confirmed significantly higher rates in neutrophils from exposed mice compared to controls (Fig. 3C).

**Fig. 4:**
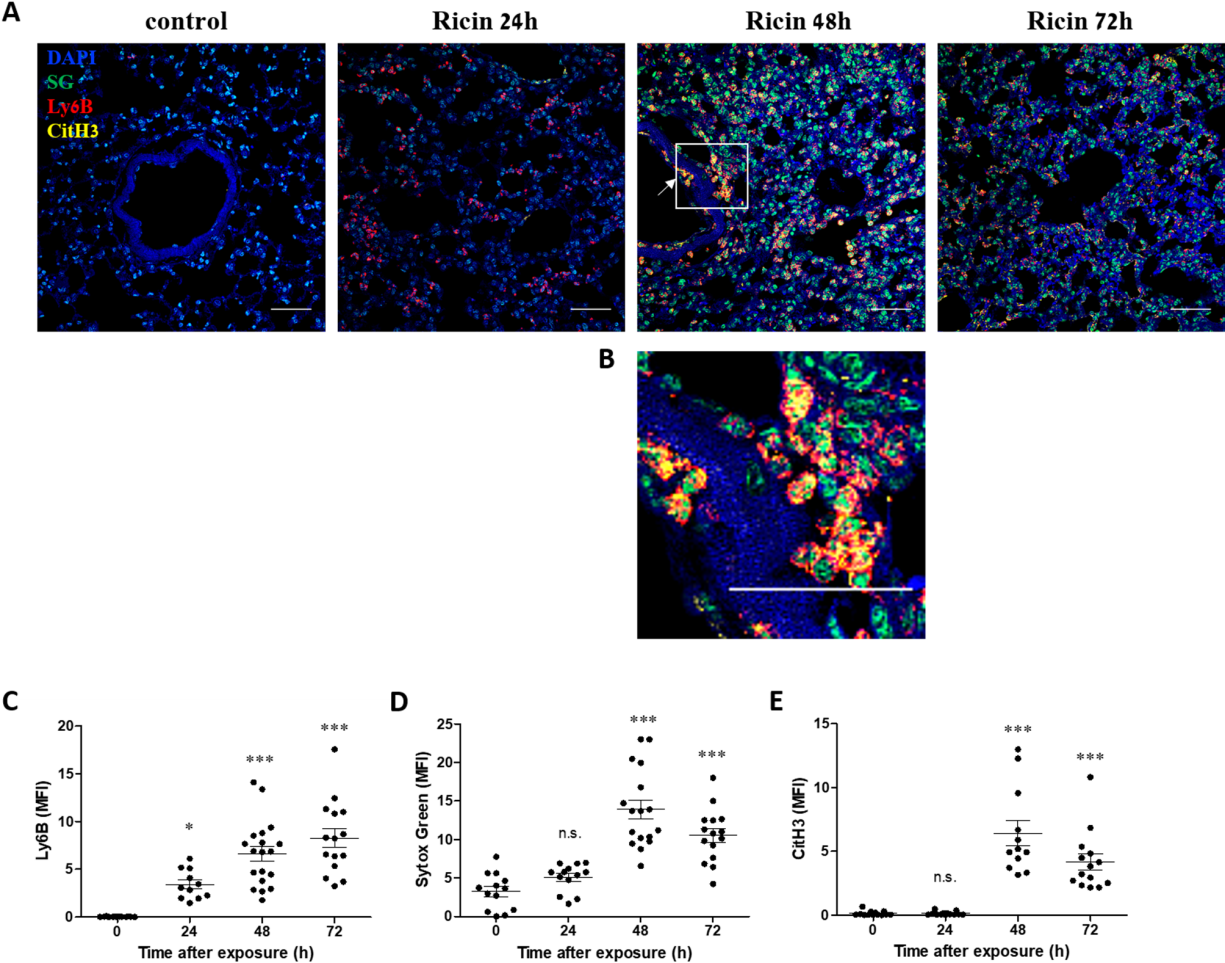
In vivo NETosis in the lungs of mice following ricin exposure**.** **A** Representative images of lung tissue sections stained for neutrophils (Ly6B, red), citH3 (yellow), extracellular DNA (Sytox Green, green), and nuclei (DAPI, blue) at different time points from naïve control mice and mice intranasally exposed to 9.6 µg/kg (2LD50) ricin. NETosis was indicated by the presence of extracellular chromatin colocalized with citH3 staining, increasing over time post-exposure. At 48 h post-exposure, an arrow shows NETosing neutrophils while their extravasation through blood vessel into the lung. Scale bar: 50 μm. **B** Enlarged view of the region outlined by rectangle in **A** shows neutrophils (red) positive for citH3 (yellow) and Sytox Green (green). Scale bar: 50 μm. **C–E** Quantification of mean fluorescence intensity (MFI) for Ly6B (**C**), extracellular DNA (Sytox Green) (**D**) and citH3 (**E**) in lung sections at different time points post-exposure. Data are presented as mean ± SEM (*n* = 5 per group). For each lung section, at least three nonoverlapping fields were acquired and MFI was measured. Each data point represents the MFI of an image. Statistical significance: *p < 0.05, **p < 0.01, ***p < 0.001 compared to non-exposed control mice. (n.s., not significant)

To confirm NETosis within lung tissue in vivo, mice were exposed to ricin, and lungs were harvested at 24, 48, and 72 h post-exposure for immunofluorescent analysis. This approach allowed us to visualize the presence and localization of key NETosis markers within the lung parenchyma over time. Lung sections were stained for neutrophils (Ly6B), extracellular DNA, and citH3. Control lungs contained few neutrophils (Fig. 4A, C). At 24 h post-exposure, neutrophil recruitment was evident, but NETosis had not yet occurred (Fig. 4A, C, D, E). By 48 h, numerous neutrophils exhibited colocalized extracellular DNA and citH3 staining, indicative of NETosis (Fig. 4A, B, C, D, E). Neutrophils infiltrating the lungs through blood vessels could also be observed, already undergoing NETosis during the process of migration (Fig. 4A, B). At 72 h, NETosis remained extensive, but was slightly reduced compared to 48 h (Fig. 4A, D, E). These findings align with the Western blot results (Fig. 1).

### The impact of NET degradation therapy in ricin intoxicated mice

**Fig. 5:**
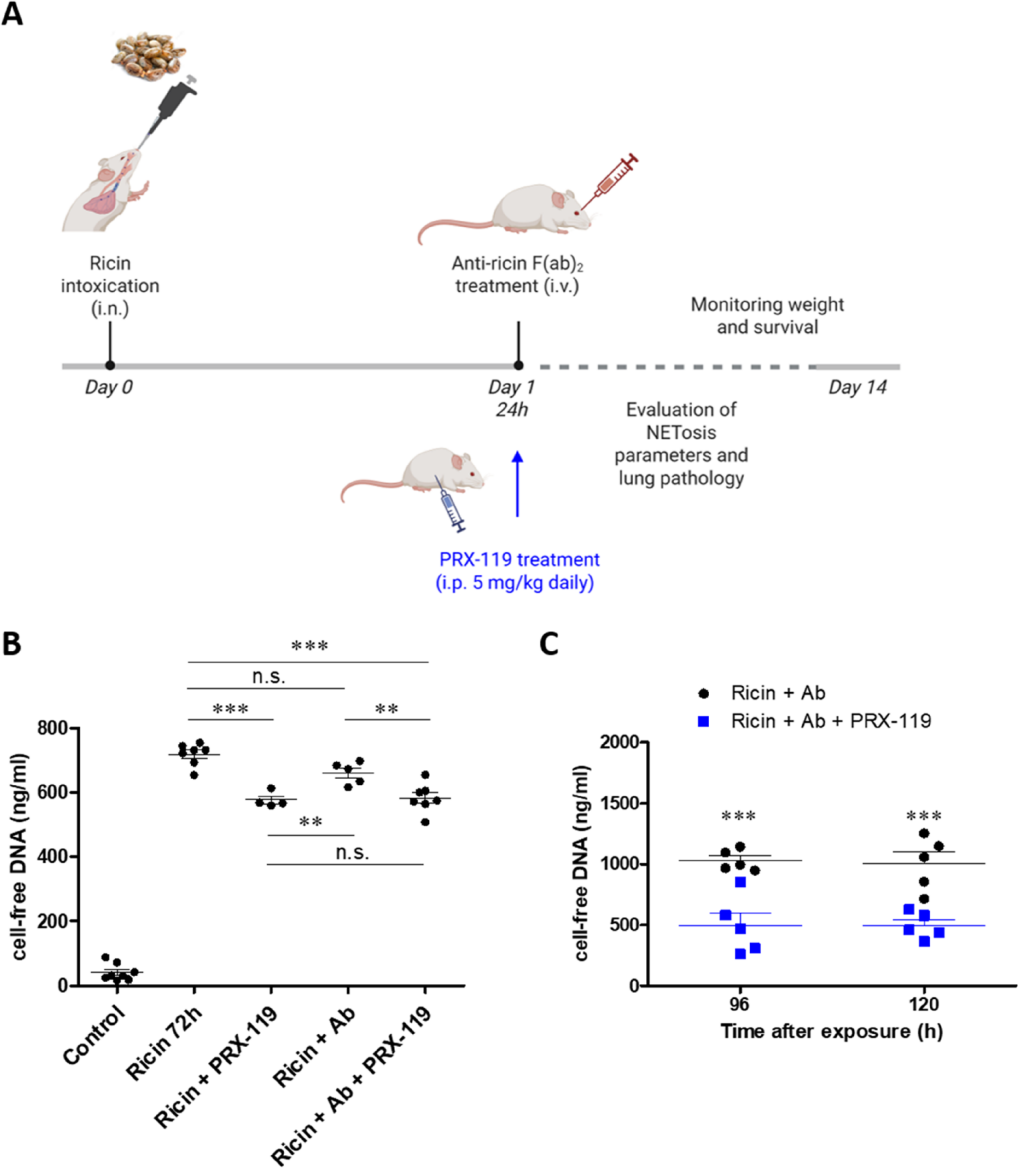
Extracellular DNA levels in BALF of mice following ricin exposure and treatment with the NET-targeting agent, PRX-119**.** **A** Schematic representation of the experimental design. Mice were intranasally exposed to 9.6 µg/kg (2LD_50_) ricin. At 24 h post-exposure, mice received either an intravenous (i.v.) injection of an anti-ricin antibody (RR003, 100 µl, single dose), daily intraperitoneal (i.p.) administration of PRX-119 (5 mg/kg), or a combination of both treatments (RR003 and PRX-119). NET degradation and lung pathology were monitored at different time-points. In addition, Body weight and survival were monitored for 14 days post-exposure. Created with BioRender.com. **B** Quantification of extracellular DNA levels in BALF of ricin-exposed mice at 72 h post-exposure, following different treatment regimens. **C** Quantification of extracellular DNA levels in BALF at 96- and 120-hours post-exposure in mice treated with anti-ricin antibody or with combination of anti-ricin-antibody and PRX-119. Each data point represents an individual mouse. Statistical significance: **p < 0.01, ***p < 0.001 comparison between antibody-only and combination treatment in C. (n.s., not significant)

Given the extensive NETosis observed in the lungs of mice that were exposed to ricin, we hypothesized that extracellular DNA release during this process contributes to lung inflammation and edema, exacerbating respiratory distress. Therefore, we evaluated whether treatment with a NET-degrading DNase I could alleviate lung pathology. For this purpose, we selected PRX-119, a PEGylated recombinant human DNase I that exhibits extended half-life in the circulation and has previously shown efficacy in sepsis mouse model of NET-related disease (Sharma et al. 2025). We employed the following experimental protocol to test the treatment: mice were intranasally intoxicated with ricin and treated at 24 h post-exposure with either PRX-119, anti-ricin antibody, or both (Fig. 5A). In our therapeutic approach, anti-ricin antibody treatment is always administered as a first-line intervention that prevents toxin binding to cells, thereby neutralizing its catalytic activity and downstream effects. Our previous studies have demonstrated that anti-ricin antibodies are essential for achieving partial survival in ricin-intoxicated animals. Notably, adjunctive therapies alone show limited or no efficacy in the absence of anti-ricin treatment. Therefore, additional targeted interventions are administered in combination with anti-ricin antibodies to enhance therapeutic outcomes (Gal et al. 2014, 2016).

At different time points post-exposure we quantified NETosis markers and NET degradation, and evaluated lung pathology (Fig. 5A). First, we examined the effect of the treatment on extracellular DNA content in the lungs. BALF was collected 72 h post-exposure from intranasally ricin-intoxicated mice, as well as from intoxicated mice treated with an anti-ricin antibody, PRX-119 as a standalone treatment, or a combination of anti-ricin antibody and PRX-119. The results indicated that in contrast to antibody treatment alone, which did not affect the high levels of extracellular DNA, PRX-119 treatment alone significantly reduced extracellular DNA levels in the lungs. Similarly, the combined treatment with the antibody and PRX-119 led to a significant decrease in extracellular DNA concentration (Fig. 5B). It is important to note that despite the significant reduction in extracellular DNA levels following PRX-119 treatment, high cell-free DNA levels remained in the lungs even 72 h post-exposure. Therefore, we measured DNA levels at later time points, 96- and 120-hours post-exposure. These measurements revealed that extracellular DNA levels continued to decline significantly following the combined treatment, compared to treatment with the anti-toxin antibody alone (Fig. 5C).

**Fig. 6:**
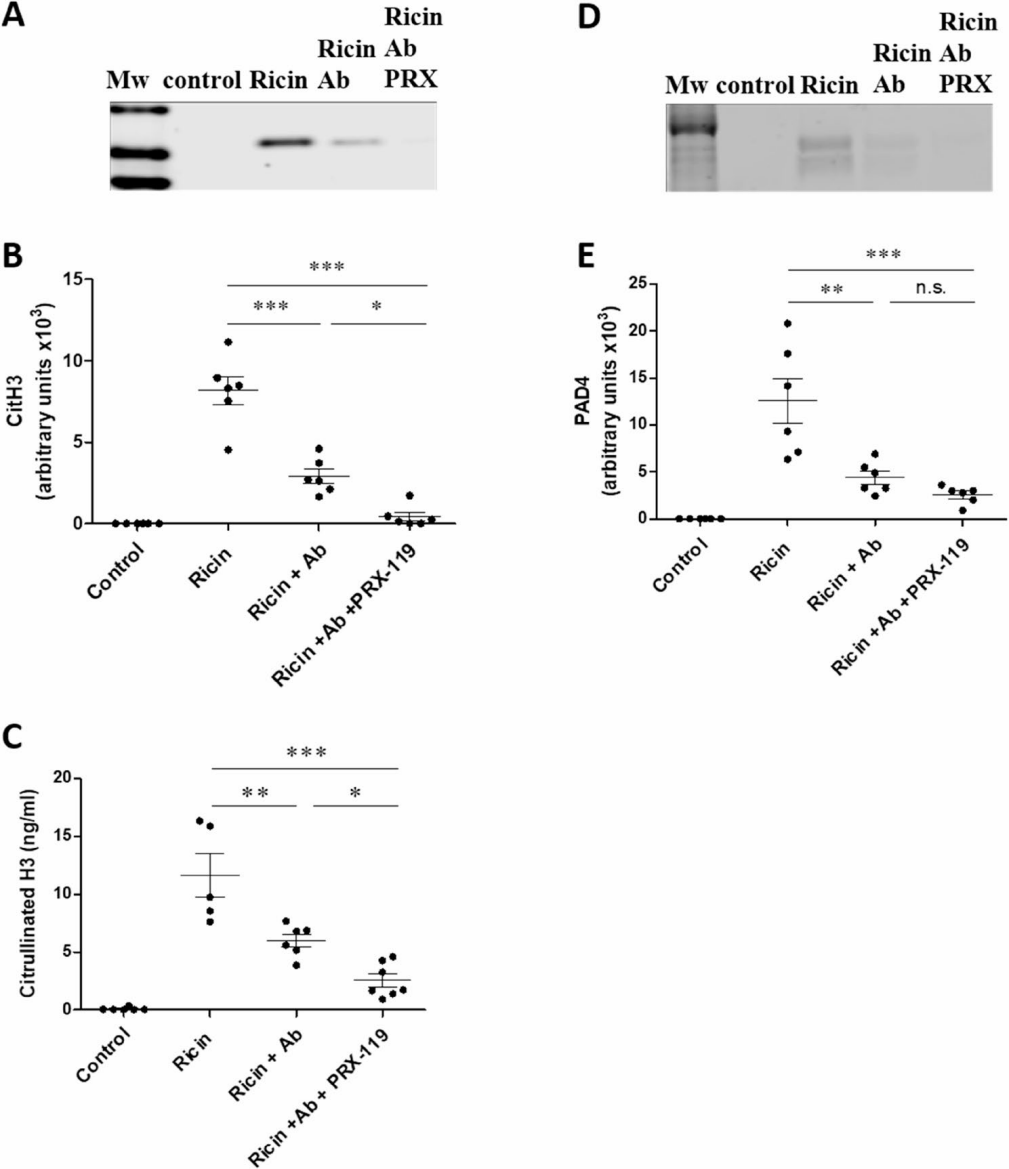
Expression levels of PAD4 and citH3 in BALF of mice exposed to ricin and treated with anti-ricin antibody and PRX-119. Western blot analysis of citH3 (**A**) and PAD4 (**D**) in BALF collected from control mice and mice intranasally exposed to 9.6 µg/kg (2LD_50_) ricin. At 24 h post-exposure, mice received either an i.v. injection of an anti-ricin antibody (100 µl, single dose) alone or in combination with i.p. administration of PRX-119 (5 mg/kg), which was continued daily until the end of the experiment. BALF was collected at 48 h post-exposure, and protein levels were analyzed. **B**,** E** Densitometric quantification of citH3 and PAD4 bands using ImageJ software. **C** Quantification of citH3 levels in BALF using a commercial ELISA assay. Data are presented as mean ± SEM (*n* = 5–7 per group, each data point represents an individual mouse). Statistical significance: *p < 0.05, **p < 0.01, ***p < 0.001. (n.s., not significant)

Next, the protein levels of key components involved in the NETosis process were analyzed by Western Blot to assess their trends following treatment. We observed a reduction in citH3 levels following antibody treatment alone, which was further enhanced following the combined therapy, reaching near-baseline levels (Fig. 6A, B). This reduction pattern was confirmed by ELISA for citH3 (Fig. 6C). PAD4 levels were also reduced following treatment with anti-ricin antibody alone, as well as in combination with PRX-119 treatment (Fig. 6D, E). These results demonstrate that the combination therapy, comprising an anti-toxin antibody and PRX-119, which degrades extracellular DNA, significantly reduced NETosis, nearly abolishing the process.

**Fig. 7:**
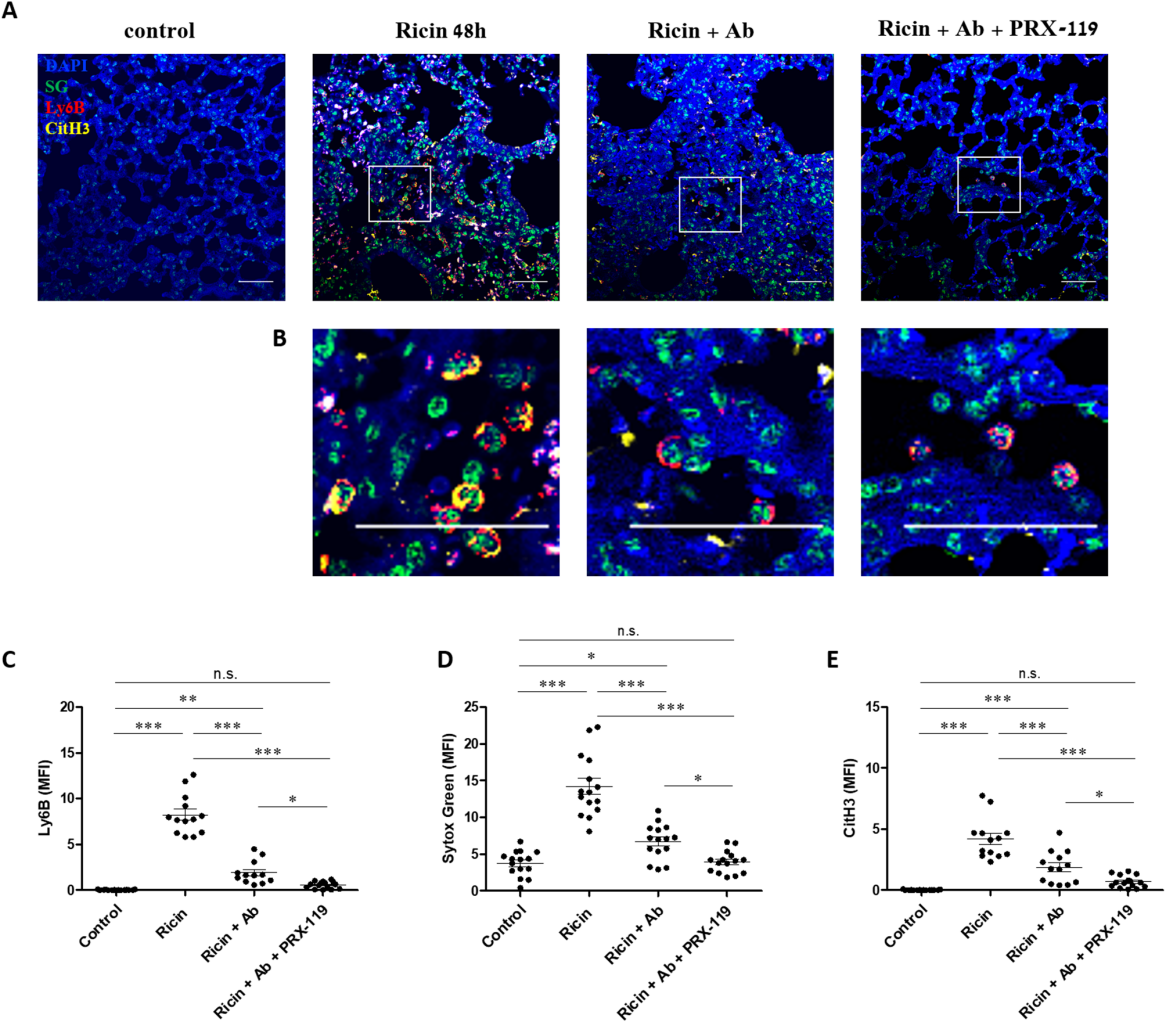
In vivo NETosis in the lungs of mice following ricin exposure and treatment with anti-ricin antibody and PRX-119. **A** Representative images of lung sections stained for neutrophils (Ly6B, red), citH3 (yellow), extracellular DNA (Sytox Green, green), and nuclei (DAPI, blue) from control mice and mice intranasally exposed to 9.6 µg/kg (2LD_50_) ricin. At 24 h post-exposure, mice received either an i.v. injection of an anti-ricin antibody (100 µl, single dose) alone or in combination with i.p. administration of PRX-119 (5 mg/kg), which was continued daily until the end of the experiment. Lungs were harvested 24 h after treatment and processed for immunofluorescence staining. Scale bar: 50 µm. **B** Enlarged views of the regions outlined by rectangles in **A** show neutrophils (red), citH3 (yellow) and Sytox Green (green) staining. Scale bar: 50 µm. **C–E** Quantification of mean fluorescence intensity (MFI) for Ly6B (**C**), extracellular DNA (Sytox Green) (**D**), and citH3 (**E**), representing NETosis intensity in lung sections at different time points post-exposure. Data are presented as mean ± SEM (n = 5 per group). For each lung section, at least three nonoverlapping fields were acquired and MFI was measured. Each data point represents the MFI of an image. Statistical significance: *p < 0.05, **p < 0.01, ***p < 0.001. (n.s., not significant)

To further monitor the effect of the above-mentioned treatments on NETosis, we analyzed lung sections 48 h post-exposure by immunofluorescence. Extensive NETosis in ricin exposed untreated mice was evident, characterized by a high number of neutrophils positive for extracellular DNA and citH3. At the same time point, a significant reduction in NETosis was observed following antibody treatment alone. In agreement with the effects on NETosis markers, an almost complete resolution of NETosis was detected in the combination treatment group (Fig. 7A, B, C, D, E).

**Fig. 8:**
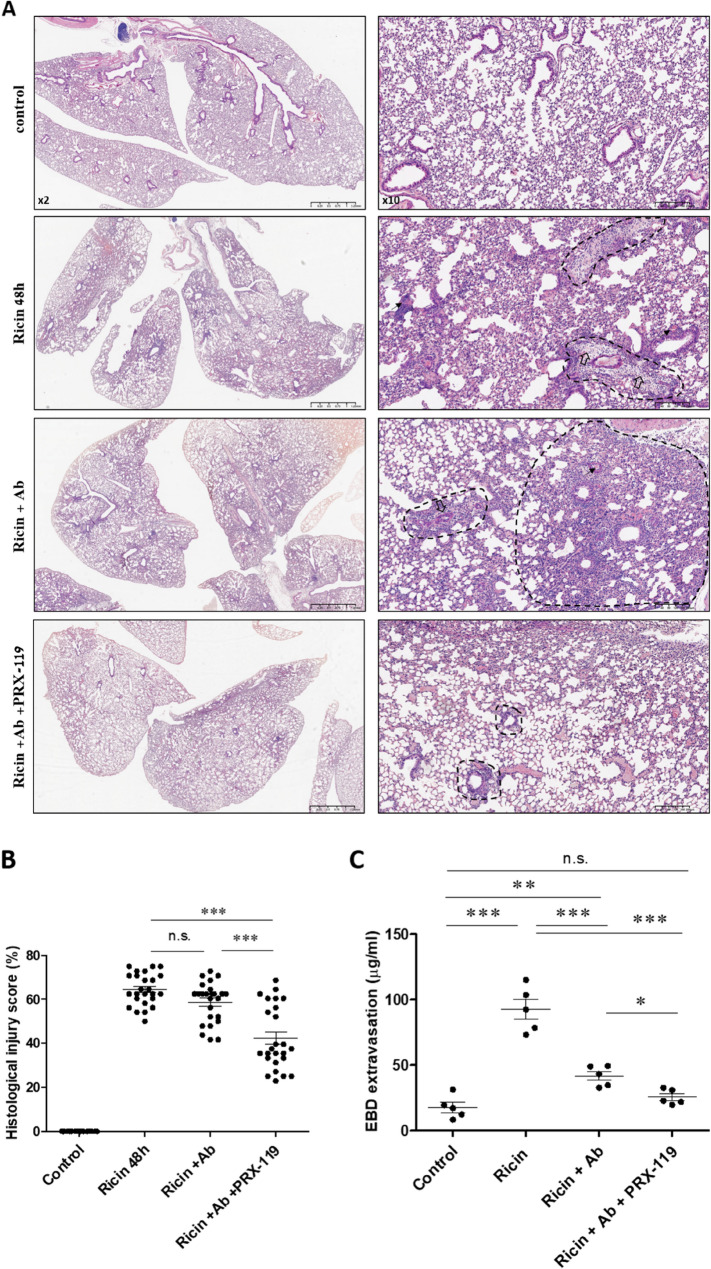
Histological analysis of mouse lungs and examination of alveolar-capillary barrier integrity following ricin exposure and treatment with anti-ricin antibody and PRX-119. **A** Representative hematoxylin and eosin (H&E) stained lung sections from mice intranasally exposed to 9.6 µg/kg (2LD_50_) ricin. At 24 h post-exposure, mice received either an i.v. injection of an anti-ricin antibody (100 µl, single dose) alone or in combination with i.p. administration of PRX-119 (5 mg/kg). Lungs were harvested 24 h after treatment and processed for histological evaluation. Scale bar: left column 1.25 mm, right column 200 μm. Dashed marking represents areas heavily infiltrated with inflammatory cells; thick arrows indicate perivascular infiltration, expansion of perivascular regions and presence of proteinaceous exudates; thin arrows indicate blood vessel congestion. **B** Quantification of lung histological injury score, assessed based on six different parameters across five randomly selected fields per lung section. Data are presented as mean ± SEM (*n* = 5 per group). Each data point represents the average histological injury score calculated from five different lung fields in each of the five analyzed lungs per group. Statistical significance: ***p < 0.001. (n.s., not significant). **C** Mice were intranasally exposed to ricin and at 24 h post-exposure, mice received either an anti-ricin antibody alone or in combination with PRX-119 that was continued daily until the end of the experiment. At 72 h post-exposure, Evans Blue dye was administered i.v. to assess alveolar-capillary barrier permeability. One hour after injection, lungs were harvested, and dye concentration was quantified as a measure of vascular leakage. Data are presented as mean ± SEM (*n* = 5 per group), with each data point representing an individual mouse. Statistical significance: *p < 0.05, **p < 0.01, ***p < 0.001. (n.s., not significant)

To evaluate the impact of treatment on lung pathology, histological analysis was performed on lung tissue sections from intoxicated and treated mice. Histological examination using hematoxylin and eosin (H&E) staining of the lungs from intoxicated mice revealed severely edematous, non-aerated lungs, heavily infiltrated with inflammatory cells and displaying increased tissue density. Focusing on perivascular regions, we observed significant neutrophil infiltration, marked expansion of these areas, and the presence of proteinaceous exudates. Pulmonary blood vessels were congested with red blood cells, surrounded by perivascular and peribronchiolar edema, and showed evidence of cellular debris indicative of cell death. Within the alveoli, prominent neutrophilic infiltration, cell death and edema were evident. The lungs exhibited extensive vascular leakage and fibrin accumulation within the alveolar airspaces. These pathological changes contrasted sharply with the normal, well-aerated lung structure observed in non-intoxicated control mice, which displayed an intact bronchiolar and alveolar architecture, contained alveolar macrophages and was devoid of neutrophil infiltration (Fig. 8A). It was observed that following antibody treatment alone, the lungs remained damaged, with poor aeration, inflammatory cell infiltration, and edematous foci (Fig. 8A). In contrast, lungs from mice that received combination therapy were better aerated, with only minimal inflammatory foci, well-preserved alveolar architecture, and very limited perivascular infiltration, appearing more similar to those of control mice (Fig. 8A). A semi-quantitative assessment of the lung histological injury score revealed that the score in the combination treatment group was significantly lower than that of the anti-ricin monotherapy group and that both treatment groups scored significantly lower than the intoxicated untreated group (42 ± 14%, 59 ± 9%, 65 ± 7% for combination treatment, monotherapy and ricin exposed untreated groups, respectively) (Fig. 8B). Increased permeability of the alveolar-capillary barrier and the influx of protein-rich fluid into the interstitium and alveolar space are hallmark features of pulmonary ricin intoxication. These processes lead to the development of pulmonary edema, respiratory failure, and ultimately, death (Gal et al. 2017). Previously, we had demonstrated that antibody treatment administered 24 h post-exposure to ricin reduced lung permeability (Sapoznikov et al. 2019). Here, we assessed whether the combined PRX-119 and antibody treatment could further mitigate the breakdown of the alveolar-capillary barrier compared to antibody treatment alone. To that end, lung permeability was assessed using the EBD Extravasation Assay. The results showed that while the increased permeability observed at 72 h post-exposure was partially reduced following antibody treatment, the combined treatment significantly improved barrier integrity compared to antibody treatment alone (Fig. 8C). These findings demonstrate that the combined treatment effectively mitigates the morphological and histological lung damage following pulmonary ricin exposure and significantly reduces the disruption of the alveolar-capillary barrier.

To evaluate the impact of PRX-119 treatment on the process of lung recovery and the reduction of inflammatory markers following ricin exposure, we examined changes in various pro- and anti-inflammatory cytokines. Cytokine levels were measured in the BALF of mice exposed to ricin and treated with either PRX-119 or anti-ricin antibody alone, or a combination of both therapies. We found that IL-6, the main pro-inflammatory cytokine which is elevated after ricin exposure (Gal et al. 2014, 2016), remained unaffected by PRX-119 treatment. However, antibody treatment against ricin significantly reduced its level. In agreement with this finding, the combined treatment did not provide any additional benefit in lowering this pro-inflammatory cytokine (Supplementary Fig. 1 A). Similarly, pro-inflammatory cytokines responsible for neutrophil recruitment to the lung tissue, such as G-CSF and KC (CXCL1), which were markedly elevated post-ricin intoxication, were significantly reduced following antibody treatment, with no further reduction observed with the combined treatment (Supplementary Fig. 1B, C). Other cytokines, including MIP-2 (CXCL2), IL-1β, MCP-1 (CCL2), VEGF, and TNF-α, which contribute to neutrophil and monocyte recruitment, increased endothelial permeability, and impaired alveolar fluid clearance, were moderately elevated after ricin exposure. PRX-119 alone had no impact on these cytokines. Unexpectedly, antibody monotherapy was found to increase the level of these cytokines in BALF, an effect which was not mitigated by the combined therapy (Supplementary Fig. 1D, E, F, G, H).

**Fig. 9:**
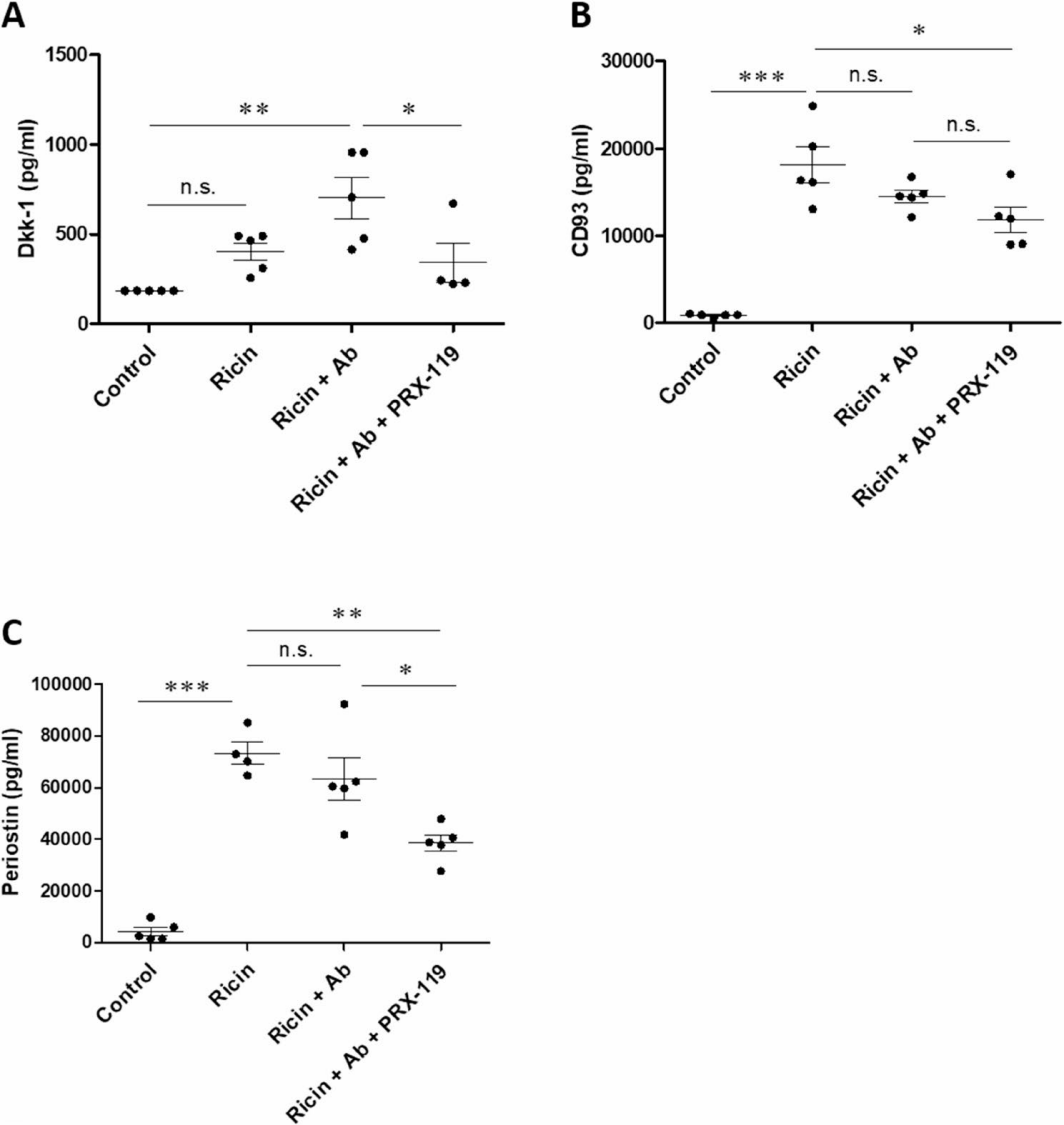
Alterations in Dkk-1, CD93 and Periostin levels in BALF of mice following ricin exposure and treatment with anti-ricin antibody and PRX-119**.** Mice were intranasally exposed to 9.6 µg/kg (2LD_50_) ricin. At 24 h post-exposure, mice received either an i.v. injection of an anti-ricin antibody (100 µl, single dose) alone or in combination with i.p. administration of PRX-119 (5 mg/kg), which was continued daily until the end of the experiment. BALF was collected at 72 h post-exposure, and protein levels were quantified using Luminex assay. Protein measurements included: **A** Dkk-1, **B** CD93 and **C** Periostin. Data are presented as mean ± SEM (*n* = 5 per group), with each data point representing an individual mouse. Statistical significance: *p < 0.05, **p < 0.01, ***p < 0.001. (n.s., not significant)

Since no significant differences were found in the levels of tested cytokines between antibody-only treatment and combined therapy, we sought to identify additional analytes that could characterize PRX-119 impact on lung recovery. For this purpose, we assembled a panel of factors involved in various pro- and anti-inflammatory processes that influence lung epithelial and endothelial cell repair, restoration of vascular leakage, reduction of pulmonary edema, fibrosis-related proteins, and markers of lung remodeling (Supplementary Fig. 2). This multi-analyte panel was assessed by Multiplex ELISA which was applied on BALF samples from three groups: ricin-exposed untreated mice, ricin-exposed mice treated with an anti-ricin antibody, and ricin-exposed mice treated with the combination of anti-ricin antibody and PRX-119 (Supplementary Fig. 3). Several analytes were identified as significantly altered following combination treatment. The protein Dkk-1 was elevated following ricin exposure, with even higher levels detected in mice that received antibody-only treatment. However, combination therapy significantly reduced Dkk-1 levels (Fig. 9A). Another protein, CD93 was found to be significantly lower in mice treated with combination therapy, whereas no substantial change was observed in the antibody-only treatment group compared to untreated ricin-exposed mice (Fig. 9B). Similarly, we found markedly elevated levels of Periostin in ricin-exposed mice, which were not significantly reduced by antibody monotherapy. In contrast, the combination therapy substantially decreased periostin levels (Fig. 9C). Altogether, these findings show that combination therapy reduced the levels of Dkk-1, CD93, and Periostin.

### NET targeted treatment contributes to protection of ricin-exposed mice

**Fig. 10:**
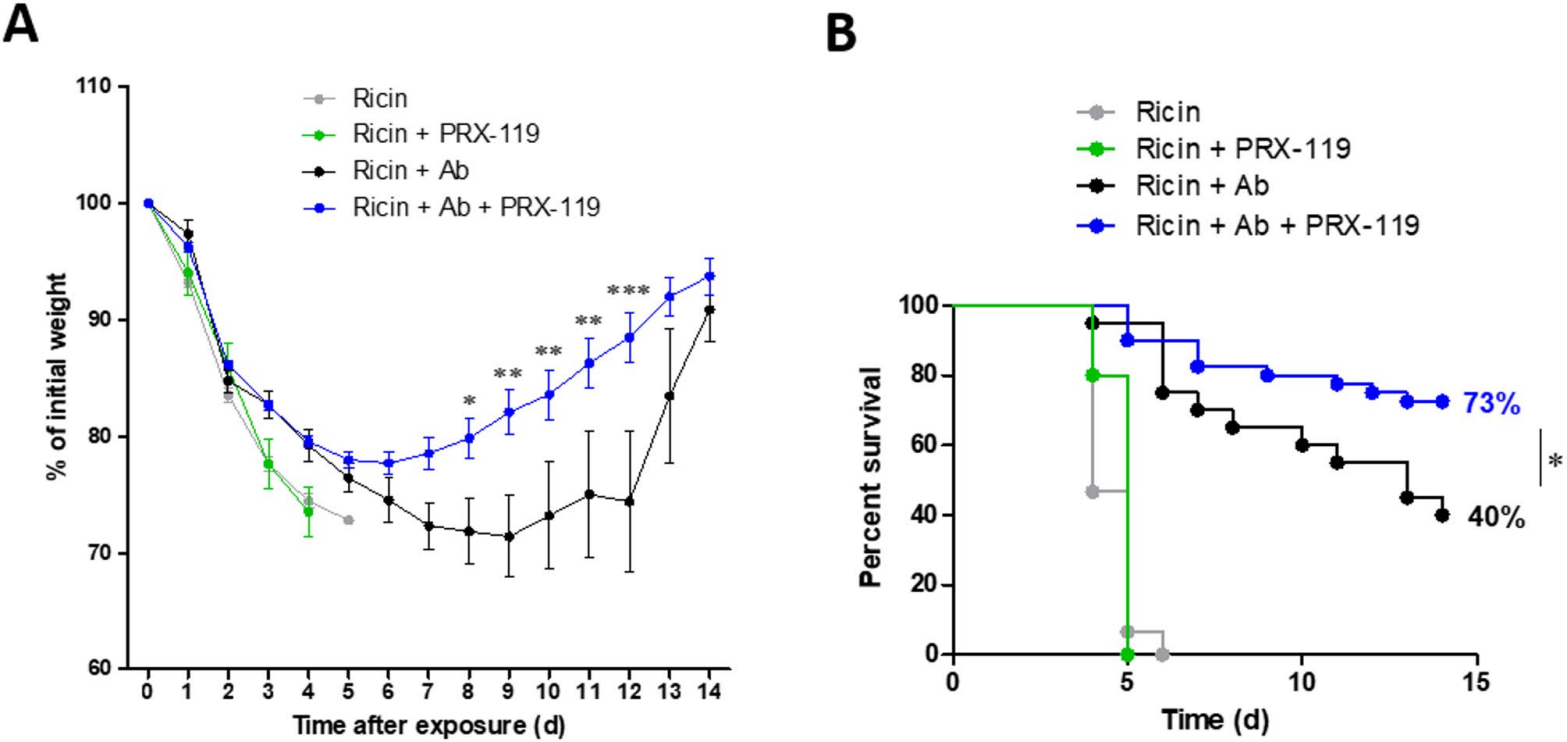
Body weight and survival of mice exposed to ricin and treated with the NET-targeting agent PRX-119. Mice were intranasally exposed to 9.6 µg/kg (2LD_50_) ricin. At 24 h post-exposure, mice received either an i.v. injection of an anti-ricin antibody or i.p. administration of PRX-119 (5 mg/kg), which was continued daily until the end of the experiment. Another group of mice received combined treatment of anti-ricin antibody and PRX-119. Body weight and survival were monitored for 14 days post-exposure. **A** Changes in body weight over the course of the experiment in different treatment groups. **B** Survival rates of mice in the different treatment groups throughout the study. Groups: ricin-exposed mice (n = 15), ricin + anti-ricin antibody (n = 15), ricin + anti-ricin antibody + PRX-119 (n = 40), and ricin + PRX-119 (n = 5). Statistical significance: *p < 0.05, **p < 0.01, ***p < 0.001 comparing antibody-only treatment to combination treatment with antibody and PRX-119

Having demonstrated that combined treatment with an anti-ricin antibody and PRX-119 inhibited NETosis, significantly mitigated lung damage and reduced the disruption of the alveolar-capillary barrier following pulmonary ricin exposure, we next evaluated its clinical effect in ricin-intoxicated mice. Mice were treated at 24 h post-exposure to ricin with either PRX-119, anti-ricin antibody, or with combination treatment. Weight loss and survival were monitored for 14 days. Mice treated with PRX-119 alone exhibited weight loss similar to ricin-intoxicated untreated mice and the animals in both groups succumbed to intoxication by day 4–6 (Fig. 10A, B). Animals receiving antibody treatment alone lost ~ 30% of body weight before beginning of recovery at around day 9. Interestingly, combination treatment further reduced morbidity as reflected by limited weight loss (20%) and earlier recovery beginning around day 6 (Fig. 10A). While PRX-119 alone did not improve survival, combination treatment significantly increased survival to 73%, in comparison to 40% survival following antibody treatment alone (Fig. 10B).

## Discussion

This study demonstrates, for the first time, a pivotal role of NETosis in the pathogenesis of ricin-induced acute lung injury and establishes that targeted degradation of NETs using PRX-119, a PEGylated plant-produced long-acting recombinant human DNase I, in combination with anti-ricin antibody therapy, significantly improves clinical outcomes in a murine model. We provide comprehensive evidence for NETosis in ricin-exposed lungs using molecular, cellular, and histological approaches, and show that its attenuation contributes to reduced lung damage and enhanced survival.

Previous studies have shown that neutrophilic infiltration is a hallmark of ricin intoxication and contributes to pulmonary injury (Sapoznikov et al. 2015, 2019; Gal et al. 2016). Our findings based on this knowledge by identifying NETosis, as one of the mechanistic drivers of lung pathology. The detection of elevated levels of PAD4, citH3, and extracellular DNA, alongside direct visualization of NETs in BALF and lung sections, confirms that ricin exposure induces robust NET formation. These findings are consistent with previous reports from other models of sterile inflammation, such as TRALI (Thomas et al. 2012), lung ischemia-reperfusion injury (LIRI) (Sayah et al. 2015) and ventilator-induced lung injury (VILI) (Yildiz et al. 2015), in which NET formation has been implicated. In addition to DAMPs that activate neutrophils, pro-inflammatory cytokines are known to play a role in initiating and amplifying NET formation. Cytokines such as TNF, IL-1β, IL-12, and type I interferons have been shown to induce NETosis (Keshari et al. 2012; Moreira-Teixeira et al. 2020). In the context of pulmonary ricin intoxication, elevated levels of these cytokines have been reported (Gal et al. 2016; Korcheva et al. 2007; Wong et al. 2007; Falach et al. 2021), suggesting that they may contribute to the induction of NETosis observed in this model. In the current model, NETosis peaked at 48 h post-ricin exposure, while partial decline in NETosis markers was observed in both BALF and lung tissue in vivo 72 h post-ricin exposure. Previous studies from our group have shown that by 72 h post-exposure, animals exhibit marked clinical deterioration and increased mortality (Gal et al. 2014), suggesting that the observed reduction in NETosis at this time point may be attributable to advanced pathophysiological decline.

The NETs clearance and the effective removal of extracellular DNA, enzymatic proteins and histones are necessary to maintain tissue homeostasis and prevent inflammation. Macrophages appear to play a key role in the clearance of NETs. This process involves active phagocytosis, which is facilitated by DNase activity and NET opsonization, followed by degradation within the lysosomal compartment (Haider et al. 2020; Farrera and Fadeel 2013). However, in the context of ricin-induced ARDS, NET clearance by macrophages appears to be significantly impaired. This is attributed to the high sensitivity of macrophages to ricin, leading to their rapid depletion from the pulmonary compartment following intoxication (Sapoznikov et al. 2015). The absence of functional macrophages likely contributes to the accumulation and persistence of NETs, potentially exacerbating the inflammatory response and tissue injury. Our findings indicate that monotherapy with an anti-ricin antibody provides only partial protection, as it fails to effectively resolve NET-mediated tissue damage, reduce extracellular DNA accumulation, or fully restore normal lung architecture. Moreover, the extracellular DNA detected in the lungs of ricin-exposed mice likely originates from several sources, including DNA fragmentation resulting from ricin’s direct cytotoxic effects, inflammation-induced cell death, and the release of NETs. Given this multifactorial contribution to extracellular DNA burden and its pathological consequences, we pursued a therapeutic approach targeting NET degradation through DNase I administration. To enhance the efficacy of this strategy, we utilized PRX-119, an investigational advanced DNase I - based agent, designed to extend systemic circulation time. This advanced therapy may be advantageous over conventional DNase molecules in degrading NETs, more efficient within the inflammatory lung microenvironment, thereby supporting more effective resolution of NET-associated damage. Indeed, we show that combined therapy with PRX-119, not only significantly decreased NETosis markers (citH3 and PAD4), but also improved lung histopathology, reduced alveolar permeability, and nearly doubled survival rates compared to antibody monotherapy. These findings are consistent with prior studies in other NET-related diseases, where degradation of extracellular DNA led to improved barrier integrity, mitigated edema and reduced inflammation (Thomas et al. 2012; Papayannopoulos et al. 2011; Saffarzadeh et al. 2012). An intriguing aspect of our findings is the selective impact of PRX-119 on downstream markers of lung injury and repair. While traditional inflammatory cytokines such as IL-6, G-CSF, and KC were primarily decreased by antibody treatment, combination therapy specifically reduced the levels of Dkk-1, CD93, and Periostin. Dkk-1 is secreted by platelets and promotes neutrophil infiltration into injured lung tissue by increasing the expression of ICAM-1 and VCAM-1, facilitating neutrophil extravasation through the alveolar-capillary barrier and exacerbating barrier permeability (Guo et al. 2015). CD93 is crucial for maintaining endothelial barrier integrity, promoting angiogenesis, and regulating VEGF levels. Additionally, it maintains cytoskeletal stability in endothelial cells, ensuring the stability of junction proteins that connect endothelial cells (Lugano et al. 2023). Periostin is secreted by fibroblasts, endothelial, and epithelial cells in response to lung epithelial injury and is implicated in pathological fibrosis. High periostin levels are correlated with impaired pulmonary function and reduced survival rates (Izuhara et al. 2016). These results suggest that NET degradation has a broader regulatory effect on the tissue microenvironment, beyond acute immune modulation.

Strengths of the study include the multi-level experimental design, combining protein analysis, imaging, functional assays, and survival endpoints. Importantly, the use of a clinically relevant therapeutic window (24 h post-exposure) enhances translational relevance. Furthermore, the use of both BALF and lung parenchyma analysis allowed for robust validation of NETosis across lung compartments. However, several limitations should be acknowledged. While NETs were shown to drive pathology, we did not genetically manipulate key NETosis mediators (e.g., PAD4 knockout), which would provide stronger mechanistic evidence. Additionally, NET inhibition rather than digestion also appears to be a promising approach to prevent ricin-induced acute lung injury, as indicated by an administration of Disulfiram, an FDA-approved gasdermin D inhibitor for the treatment of alcohol abuse, prevented signs of lung injury (Adrover et al. 2022). Gasdermin D forms pores in the nuclear plasma membrane, which are critical for NET formation (Sollberger et al. 2018). Lastly, while lung pathology and survival were assessed, the long-term functional recovery of lung tissue (e.g., gas exchange capacity or fibrotic remodeling) was not evaluated.

## Conclusions

This study identifies NETosis as a significant pathogenic mechanism in ricin-induced ARDS and establishes the efficacy of a novel combination therapy targeting both the toxin and its downstream inflammatory sequelae. Our findings highlight the therapeutic potential of combining an advanced NET-degrading agent, specifically PRX-119 with toxin-neutralizing antibodies as an effective strategy to attenuate acute lung injury and improve clinical outcomes.

## Supplementary information

Supplementary material 1.

Supplementary material 2.

Supplementary material 3.

## Data Availability

No datasets were generated or analysed during the current study.

## Abbreviations

NE: Neutrophil elastase
NETs: Neutrophil extracellular traps
BALF: Bronchoalveolar lavage fluid
PAD4: Peptidyl arginine deiminase 4
citH3: Citrullinated histone H3
MPO: Myeloperoxidase
PAMPs: Pathogen-associated molecular patterns
DAMPs: Damaged-associated molecular patterns
ROS: Reactive oxygen species
COPD: Chronic obstructive pulmonary disease
ALI: Acute lung injury
ARDS: Acute respiratory distress syndrome
MMPs: Matrix metalloproteinases
G-CSF: Granulocyte colony-stimulating factor
KC: Keratinocyte chemoattractant
MIP-2: Macrophage inflammatory protein-2
IL-1β: Interleukin-1β
IL-6/12: Interleukin-6/12
MCP-1: Monocyte chemoattractant protein
VEGF: Vascular endothelial growth factor
TNF-α: Tumor necrosis factor-α
EBD: Evans Blue dye
H&E: Hematoxylin and eosin
TRALI: Transfusion-related acute lung injury
LIRI: Lung ischemia-reperfusion injury
VILI: Ventilator-induced lung injury
