# Supplementary material for: NET degradation attenuates ricin-induced acute lung injury and protects mice from ARDS
Source: Mol Med. 2025 Sep 29;31:304. doi: 10.1186/s10020-025-01370-8 (PMC12481763; doi:10.1186/s10020-025-01370-8)
Supplement: Supplementary file 1 — Supplementary material 1. [file 10020_2025_1370_MOESM1_ESM.docx]

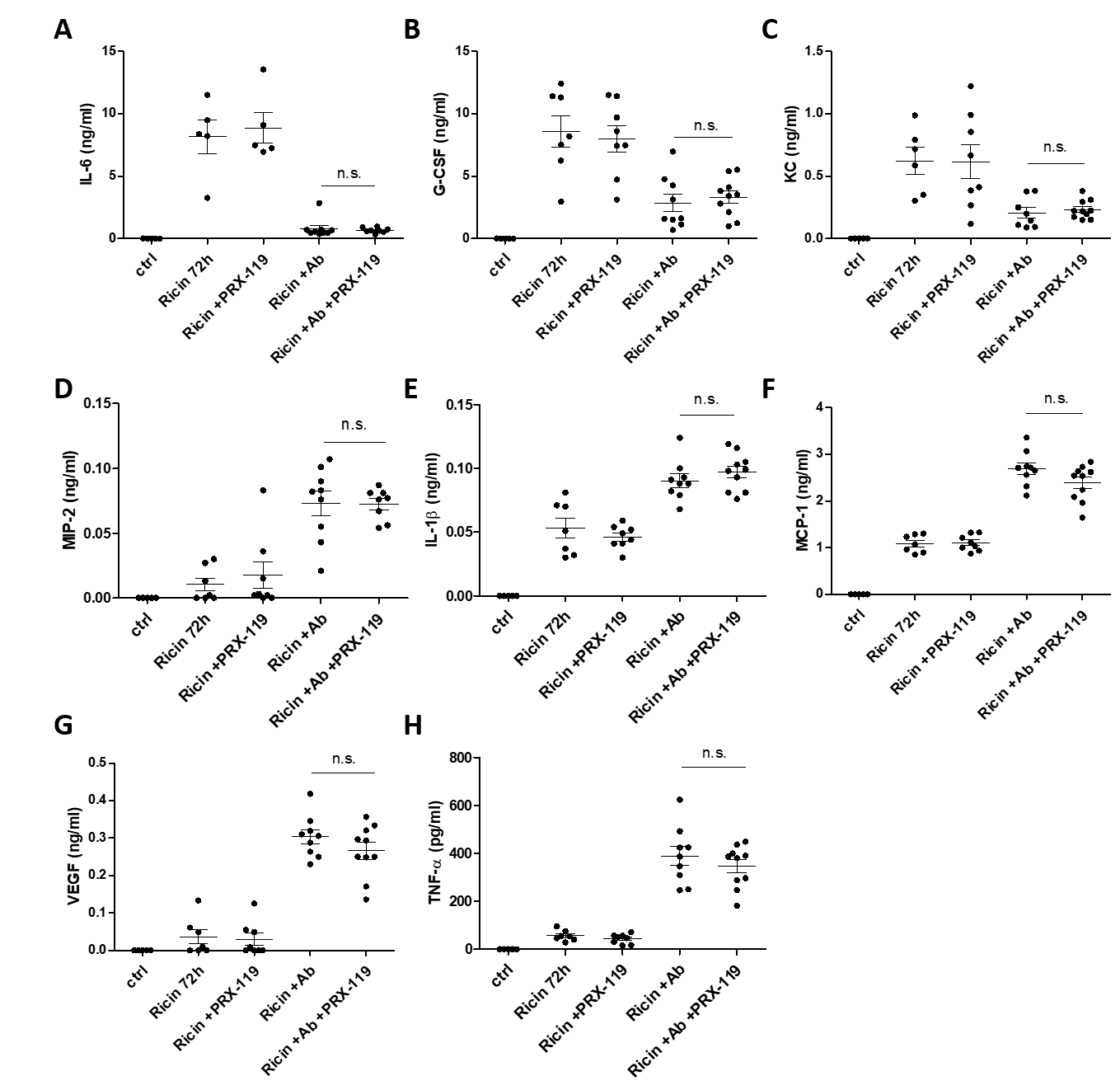


**Supplementary Figure 1. Changes in pro- and anti-inflammatory cytokine levels in BALF of mice following ricin exposure and treatment with anti-ricin antibody and PRX-119.** Mice were intranasally exposed to 9.6 µg/kg (2LD_50_) ricin. At 24 hours post-exposure, mice received either an i.v. injection of an anti-ricin antibody (100 µl, single dose) or i.p. administration of PRX-119 (5 mg/kg), which was continued daily until the end of the experiment. Additional group received a combined treatment of antibody and PRX-119. BALF was collected at 72 hours post-exposure, and cytokine levels were quantified. Cytokine measurements included: (**A**) IL-6, (**B**) G-CSF, (**C**) KC, (**D**) MIP-2, (**E**) IL-1β, (**F**) MCP-1, (**G**) VEGF, and (**H**) TNF-α. Data are presented as mean ± SEM (n = 5–10 per group), with each data point representing an individual mouse. (n.s., not significant).
