# Supplementary material for: NET degradation attenuates ricin-induced acute lung injury and protects mice from ARDS
Source: Mol Med. 2025 Sep 29;31:304. doi: 10.1186/s10020-025-01370-8 (PMC12481763; doi:10.1186/s10020-025-01370-8)
Supplement: Supplementary file 2 — Supplementary material 2. [file 10020_2025_1370_MOESM2_ESM.docx]

| C1qR1/CD93 | Plays a crucial role in maintaining endothelial barrier function and vascular integrity, promotes angiogenesis and tissue remodeling |
| --- | --- |
| CCL11/Eotaxin | A pro-inflammatory chemokine that exacerbates lung injury by recruiting polymorphonuclear cells, amplifying tissue damage and promoting vascular permeability |
| CCL22/MDC | Regulates immune resolution by recruiting Tregs to the lung, dampening excessive neutrophilic inflammation and promoting tissue repair |
| Chitinase 3-like 1/YKL-40 | A pro-inflammatory and pro-fibrotic mediator in ARDS. It reflects epithelial/endothelial damage, amplifies neutrophil-driven inflammation and contributes to tissue remodeling and fibrosis |
| CXCL16 | Plays a significant role in development of ARDS, contributes to epithelial barrier dysfunction, promotes ROS production |
| HGF | Alleviates lung injury by protecting lung permeability, affects endothelial cell function and coagulation |
| IL-1α/IL-1F1 | Pro-inflammatory cytokine, contributes to the development of ARDS |
| IL-10 | Anti-inflammatory cytokine, suppresses hyperinflammation, reduces neutrophil-driven lung injury, and preserves barrier function |
| IL-13 | Anti-inflammatory cytokine, promotes M2 macrophage polarization, is associated with tissue repair and resolution of inflammation |
| MMP-9 | Gelatinase, plays a significant role in the pathogenesis ARDS by modulating inflammation, extracellular matrix (ECM) remodeling and disruption of alveolar-capillary barrier |
| Osteopontin | Osteopontin promotes inflammation, immune cell recruitment, and fibrotic remodeling in ARDS, contributing to alveolar damage and disease severity |
| Periostin/OSF-2 | Indicative of epithelial injury, common feature in respiratory disease |
| Resistin | Enhances TLR-4-induced inflammatory response, contributes to severity of ALI, promotes activation of neutrophils |
| S100A9 | Drives ARDS progression by promoting inflammation, neutrophil recruitment and alveolar-capillary barrier disruption |
| Serpin E1/PAI-1 | Exacerbates ARDS by inhibiting fibrinolysis, promoting alveolar fibrin deposition and microthrombosis, thereby worsening lung injury |
| Thrombospondin-4 | Glycoprotein, plays a role in tissue remodeling and vascular permeability |
| TIMP-1 | Modulates ARDS progression by inhibiting MMPs to limit tissue damage |
| Dkk-1 | Plays a significant role in alveolar-capillary barrier during ARDS, promotes neutrophil infiltration and adhesion molecule expression |
| EMMPRIN/CD147 | Exacerbates ARDS by inducing MMPs, promoting inflammation and increasing alveolar-capillary barrier permeability |
| LIX | Drives ARDS progression by recruiting neutrophils to the lungs, amplifying inflammation and alveolar-capillary barrier damage |

**Supplementary Figure 2. The analytes measured in BALF by Luminex Mouse Discovery Assay and their potential roles in ARDS**
