# Supplementary material for: NET degradation attenuates ricin-induced acute lung injury and protects mice from ARDS
Source: Mol Med. 2025 Sep 29;31:304. doi: 10.1186/s10020-025-01370-8 (PMC12481763; doi:10.1186/s10020-025-01370-8)
Supplement: Supplementary file 3 — Supplementary material 3. [file 10020_2025_1370_MOESM3_ESM.docx]

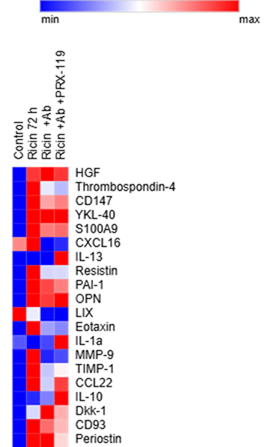


**Supplementary Figure 3. Differential analyte levels in BALF of mice following ricin exposure and treatment with anti-ricin antibody and PRX-119.** Mice were intranasally exposed to 9.6 µg/kg (2LD_50_) ricin. At 24 hours post-exposure, mice received either an i.v. injection of an anti-ricin antibody (100 µl, single dose) alone or in combination with i.p. administration of PRX-119 (5 mg/kg), which was continued daily until the end of the experiment. BALF was collected at 72 hours post-exposure, and protein levels were quantified using Luminex assay. The heatmap depicts the average analyte concentrations measured in each treatment group (n = 5 per group). No statistically significant differences were detected between the “Ricin + Ab” and “Ricin + Ab + PRX-119” groups for any analytes, except for Dkk-1, CD93 and Periostin, as shown in Figure 9.
